# Automatic Quantification of Atmospheric Turbulence Intensity in Space-Time Domain

**DOI:** 10.3390/s25051483

**Published:** 2025-02-28

**Authors:** Damián Gulich, Myrian Tebaldi, Daniel Sierra-Sosa

**Affiliations:** 1 Centro de Investigaciones Ópticas (CONICET La Plata-CIC-UNLP), La Plata 1897, Argentina; dgulich@ciop.unlp.edu.ar (D.G.); myrianc@ciop.unlp.edu.ar (M.T.); 2 Departamento de Ciencias Básicas, Facultad de Ingeniería, Universidad Nacional de La Plata (UNLP), La Plata 1900, Argentina; 3 Electrical Engineering and Computer Science, The Catholic University of America, Washington, DC 20064, USA

**Keywords:** atmospheric turbulence, deep learning, space-time analysis, video analysis, turbulence intensity quantification

## Abstract

Quantifying atmospheric turbulence intensity is a challenging task, particularly when assessing real-world scenarios. In this paper, we propose a deep learning method for quantifying atmospheric turbulence intensity based on the space-time domain analysis from videos depicting different turbulence levels. We capture videos of a static image under controlled air turbulence intensities using an inexpensive camera, and then, by slicing these videos in the space-time domain, we extract spatio-temporal representations of the turbulence dynamics. These representations are then fed into a Convolutional Neural Network for classification. This network effectively learns to discriminate between different turbulence regimes based on the spatio-temporal features extracted from a real-world experiment captured in video slices.

## 1. Introduction

Atmospheric turbulence, characterized by fluctuations in air temperature and pressure, significantly impacts the propagation of electromagnetic waves, causing variations in the refractive index of air and leading to wavefront degradation [1]. This phenomenon affects the performance of numerous communication and imaging systems, including ground-based telescopes and optical communication links, that operate at various wavelengths, including visible and infrared spectrum [1,2].

Atmospheric turbulence, characterized by fluctuations in temperature, pressure, and refractive index, has long been a challenge in various optical applications, including astronomical imaging, free-space optical communication, and remote sensing. Quantifying turbulence strength is critical for mitigating its impact, and several methods have been developed to achieve this [1].

The refractive index structure constant, $C_{n}^{2}$, serves as a key parameter for quantifying atmospheric turbulence intensity, offering valuable insights for optimizing the performance of optical systems [1]. Traditionally, dedicated instruments are employed to measure this parameter [3,4,5]; however, these instruments can be expensive, difficult to implement, or not easily available. As a consequence, obtaining high-resolution, continuous measurements of $C_{n}^{2}$ with more accessible techniques becomes relevant [6,7,8].

Historically, turbulence quantification has relied on physical instruments designed to measure the refractive index structure constant ($C_{n}^{2}$) and its integrated effects on the propagation paths. Some of these approaches include the following [1,5]:

- Scintillometers: Measure intensity fluctuations in a light beam passing through turbulence. Provide path-integrated turbulence strength but may lack spatial resolution.
- Sonic Anemometers: These devices measure the three-dimensional sound velocity, which is directly related to variations in air temperature.
- Angle-of-Arrival (AoA) Methods: Track the deflection of wavefronts due to turbulence. Require high-precision tracking equipment and are sensitive to sensor alignment.
- Shack–Hartmann Sensors: Analyze wavefront distortions using lenslet arrays.
- Differential Methods [5,9]: Image motion caused by turbulence can be quantified using differential motion variance measurements.

These approaches are widely used in adaptive optics and optics communications but may

(a) require specialized optical setups;
(b) have limitations, particularly in terms of cost, accessibility, and the need for specialized equipment;
(c) require complex post-processing techniques.

The impact of atmospheric turbulence extends beyond spatial distortions in the final image. These dynamic fluctuations that evolve over time require methods that can capture not only the spatial variations but also the temporal evolution of the turbulence effects. We may, therefore benefit, from studies carried out in the space-time plane [10].

### Video-Based Machine Learning for Turbulence Quantification in Space-Time Domain

Recent research has explored the application of deep learning tools for studying atmospheric turbulence [11,12,13,14,15,16,17].

Recent advances in deep learning for turbulence mitigation highlight the growing importance of data-driven methods in atmospheric optics and imaging. Several studies have explored the potential of deep neural networks and Convolutional Neural Networks to enhance image reconstruction and turbulence quantification [18,19,20,21,22]. These works collectively demonstrate that machine learning-driven turbulence analysis is a rapidly evolving field, paving the way for more real-time, adaptive, and physics-informed solutions in atmospheric imaging and optical communication. However, turbulence strength quantification with deep learning techniques is a relatively new field [23,24,25].

Our approach, as will be described in the following Section, differs from traditional image-based methods by leveraging deep learning to automatically extract turbulence intensity from video sequences. Instead of computing differential motion statistics or wavefront distortions explicitly, we train a Convolutional Neural Network (CNN) to classify turbulence intensity based on spatio-temporal patterns present in the video data.

This work proposes a novel approach to determine $C_{n}^{2}$ using a categorical classification based on deep learning. Our video-based approach [7,8] leverages readily available data from inexpensive sensors, offering a cost-effective and more accessible alternative to traditional methods. Furthermore, this method focuses on the horizontal ground view scenario, where the effects of turbulence are particularly pronounced due to the extended propagation path [26].

## 2. Materials and Methods

### 2.1. Experimental

**Figure 1:**
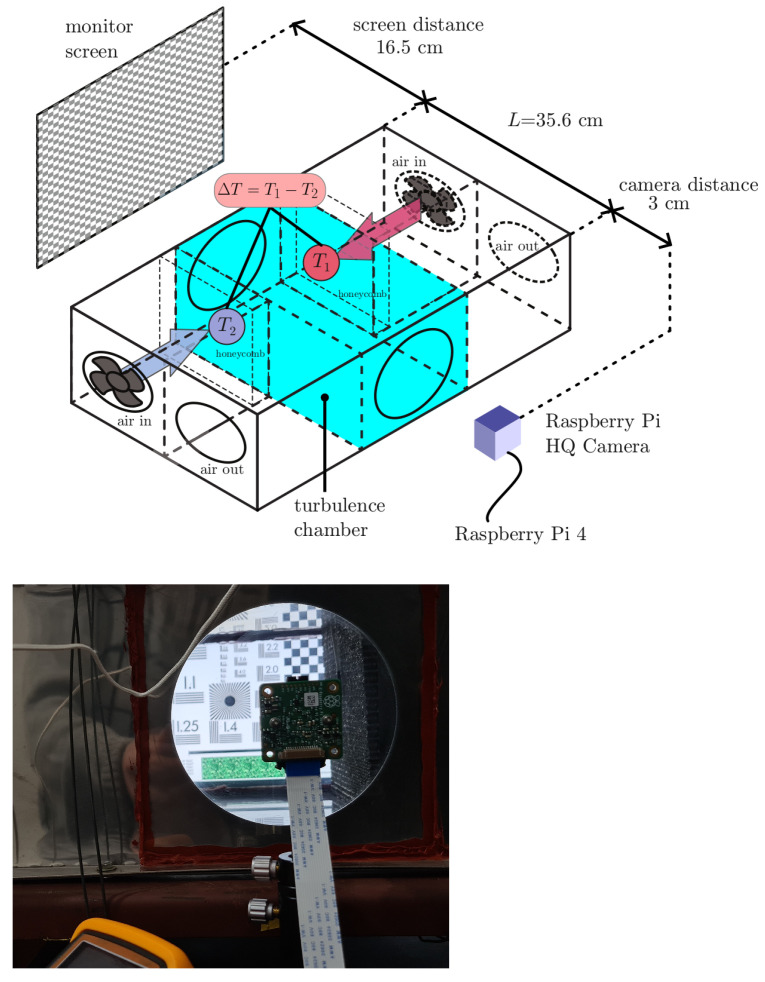
(**Above**) Experimental setup: The checkerboard (left) represents the static image located at one side of the turbulator, the camera is located on the opposite end. The monitor screen is located at 16.5 cm from the entrance window of the turbulence chamber; propagation length of light inside the the turbulator is L=35.6cm, and the camera is 3 cm away from the exit window of the unit. (**Below**) Setup detail, as seen from the camera side.

A simple image propagation experiment was performed under controlled conditions, a fixed pattern was displayed on a standard LED monitor followed by a region with artificial turbulence, and then the image on the monitor was focused into a camera [7,8], as shown in Figure 1. To have a fully developed inertial turbulence with stable and statistically repeatable conditions, we employed a laboratory turbulence chamber (*turbulator*) [27,28]. To simulate atmospheric turbulence, two air flows at different temperatures were forced to collide in the chamber, producing an isotropic mixture of hot and cold air. Light from the objective propagated through L∼ 0.35 m of turbulence in the mixing chamber. In this apparatus, the turbulence characteristics were only due to the temperature difference ($\Delta T=T_{1}-T_{2}$) between the cold source (room temperature) and the hot source. By increasing the temperature of the hot source, different turbulence intensities could be achieved. The turbulator offers the advantage of a stable behavior for any turbulence intensity value in its operating range, adjustable by the user simply by setting the temperature difference. The intensity of the turbulence, quantified by the structure constant of the refractive index ($C_{n}^{2}$), was previously characterized for the apparatus as a function of the temperature difference ΔT between the cold and hot source [29,30]. Experiments were carried out with 15 temperature differences ranging from 2.6 C to 106.2 C, all with characterized $C_{n}^{2}$ values.

The turbulator used follows the design of Keskin, Jolissaint, and Bradley [28,31,32,33]. The turbulator was characterized using the angle of arrival (AoA) method, which is a well-known technique to analyze optical turbulence [29,34]. This approach assumes a single and uniform turbulence layer (as is the case in the experimental device), enabling the extraction of key turbulence parameters such as the refractive index structure constant ($C_{n}^{2}$), the inner scale ($\ell_{0}$), and the outer scale ($L_{0}$). In this particular model, the inner scale is found to be ℓ∼1mm and the outer scale is $L_{0}∼16\;\mathrm{cm}$ (this last being, as expected, compatible with the size of the turbulence chamber), while the $C_{n}^{2}$ values are determined for each ΔT value. It should be noted that the characterization technique [28,31,32,33] is actually adjusted in terms of $C_{n}^{2}\Delta h$, where Δh is the propagation distance that, being constant in the device, enables it to calculate $C_{n}^{2}$. This feature allows for experiments representative of lower turbulence intensities with longer propagation paths, assuming that the characterization hypotheses, such as the layer of uniform turbulence intensity, are fulfilled along said propagation path [27,33,34].

In this unit (see Figure 1), the temperature $T_{2}$ is the ambient temperature of the laboratory (24 C), while the temperature of the hot source ($T_{1}$) is regulated by a variable (and measured) alternating current flowing through an internal resistor behind the corresponding honeycomb. A two-input thermocouple gives a direct reading of the value of ΔT. Knowing the previous characterization of the device based on the AoA method (as mentioned above), $C_{n}^{2}$ values are calculated from the ΔT set obtained in this work. The correspondence between ΔT and $C_{n}^{2}$ is shown in Table 1.

The capture equipment consisted of inexpensive and off-the-shelf components:

1. Raspberry Pi High-Quality Camera module (Sony IMX477R stacked back-illuminated sensor, 12.3 megapixels, 7.9 mm sensor diagonal, 1.55 µm × 1.55 µm pixel size). The sensor module was located 3 cm away from turbulator window, as shown in Figure 1.
2. The mage on the sensor was focused with a 6 mm 3 MP Wide-Angle Lens for Raspberry Pi HQ Camera—3 MP (6 mm focal length; F1.2 aperture; 63 degrees field angle).
3. Raspberry Pi 4 (Model 4b) board was used to record the videos.

For each intensity of turbulence, a capture of ∼56 s at 24 fps and a resolution of 640 × 480 was achieved with the ‘raspividyuv’ command with the ‘–nopreview’ option for performance. This camera model records in ‘yuv’ format without compression, from which the luminance was extracted for analysis (with greater contribution from the green of the Bayer filter, ∼550 nm).

**Figure 2:**
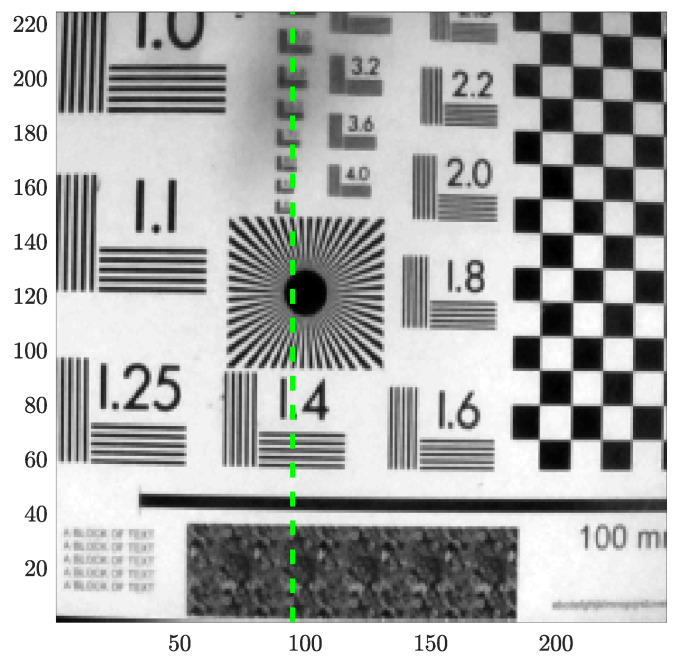
ROIimage of the target (225 × 244 pixels). The column whose temporal evolution is shown in green is in Figure 4.

**Figure 3:**
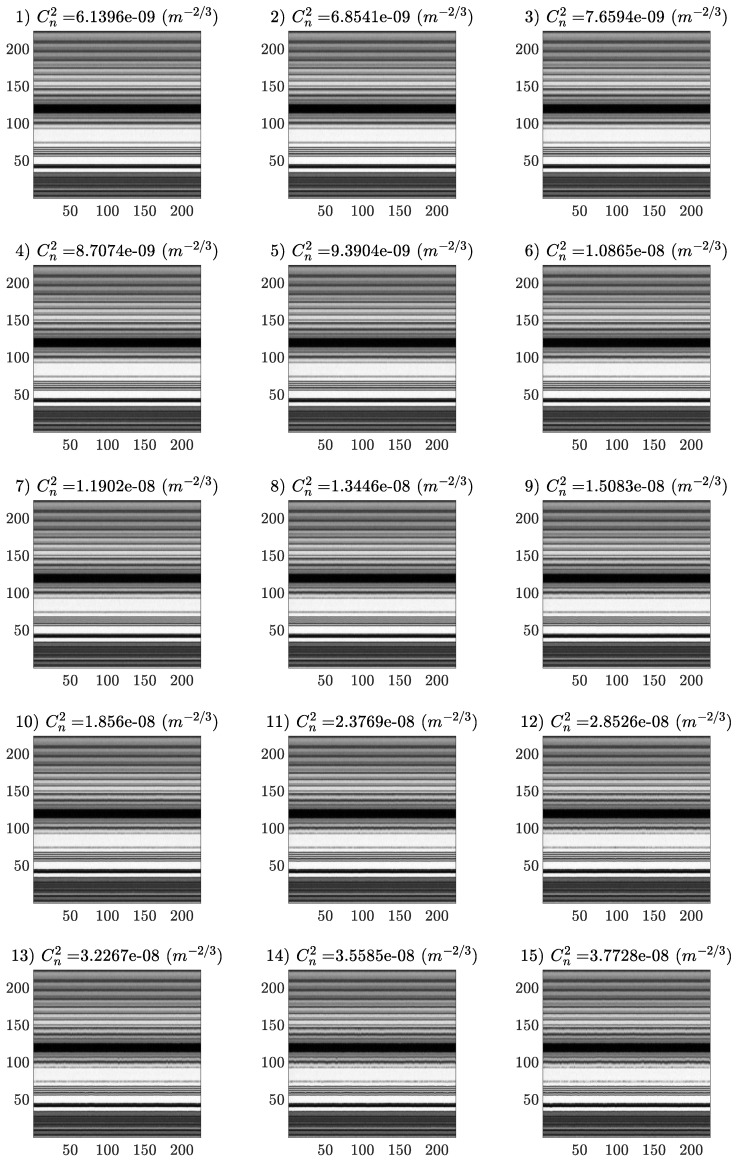
Temporal evolution (constructed over the first 225 frames) of the column indicated in the sample image (Figure 2) for all increasing turbulence intensities used for model training.

The Region of Interest (ROI) was 225 × 244 pixels; images were converted to uncompressed gray-scale images (uint8), and a representative image depicting the analyzed pattern is shown in Figure 2. Temporal evolution images (Figure 3) were generated for each turbulence intensity, as follows:

- every turbulence intensity video capture originated a set of images with the corresponding label (‘03’, …, ‘17’), giving a total of 15 sets;
- we extracted only information within the ROI from each video capture (225 × 244 × 1346 uint8 array);
- the evolution over time of each column in the ROI was extracted from the previous array as 225 × 1346 uint images (244 columns);
- every image of column temporal evolution from the previous step was split in order to produce 225 × 225 pixels uint8 images (each one covering ∼9 s of temporal evolution).

This produced 15 sets of 1220 images in each set, giving a total of *N* = 18,300 images for the analysis. No set augmentation techniques were used, as discussed in Section 4.1.

**Figure 4:**
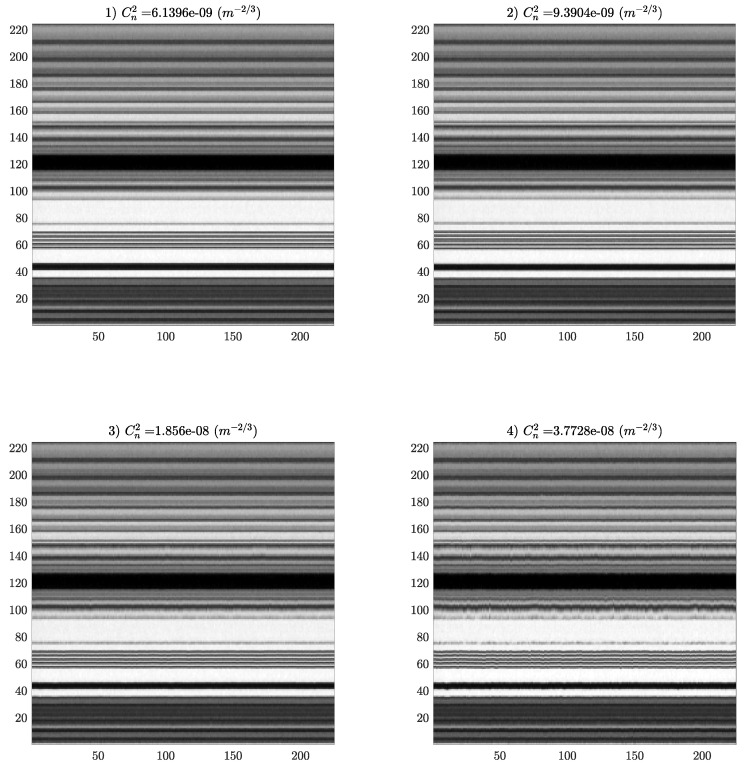
Temporal evolution (constructed over the first 225 frames) of the column indicated in the sample image (Figure 2) for 4 selected increasing turbulence intensities representative of the range studied. In this scale, the effect of turbulence increase on temporal evolution may be better observed.

In Figure 4, we show four representative images of the turbulence range studied, in which it can be seen that the fluctuation over time (horizontal axis) increases with the intensity of the turbulence. By analyzing the evolution images, it is possible to characterize the intensity of the turbulence.

### 2.2. Training Model

We present a Convolutional Neural Network (CNN) architecture for image classification tasks. The model consists of sequential layers designed to efficiently learn and classify images. The three-layer CNN architecture was chosen to balance the complexity and generalization of the model, ensuring an effective classification of turbulence while preventing overfitting. The progressive feature extraction approach, which starts with shallow convolutions (8 filters) for basic spatial features, followed by deeper layers (16 and 32 filters) for complex turbulence patterns, allows the network to capture essential image distortions while maintaining computational efficiency. Alternative architectures, such as deeper CNNs, residual networks (ResNets), and Vision Transformers (ViTs), were considered; however, the limited dataset size and the need for real-time processing favored a lightweight, interpretable model.

**Figure 5:**
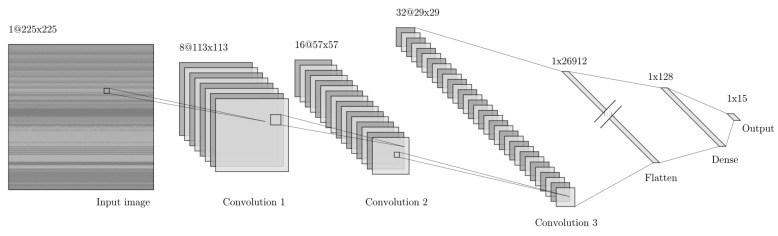
Convolutional Neural Network (CNN) architecture for image classification used.

The CNN model was implemented in Keras [35], a widely used deep learning library. The architecture (see Figure 5) is outlined as follows:

1. Input Layer: Accepted images of size 225 × 225 pixels.
2. Convolutional Layers: Three convolutional layers were employed, each applying 3 × 3 filters with Rectified Linear Unit (ReLU) activation. Stride of (2, 2) and ’same’ padding were used to reduce spatial dimensions by half at each layer.
3. Flatten Layer: Converted the output of convolutional layers into a 1D array.
4. Fully Connected Layers: Two dense layers were added, consisting of 128 neurons with ReLU activation in the first layer and a softmax activation layer with N=15 neurons (*N* being the number of output categories) in the second layer.
5. Compilation: The model was compiled using the Adam Optimizer with categorical cross-entropy loss. Performance was evaluated using two metrics [36,37]:
  (a) accuracy (Number of correct predictions/Total number of predictions).
  (b) cross-entropy loss function for multi-class classification, defined as $-\frac{1}{n}\sum_{i=1}^{n}\sum_{c=1}^{N}y_{ic}\log\left({\hat{y}}_{ic}\right)$, where $y_{ic}$ represents the true label (either 0 or 1) of the *i*-th sample for the *c*-th class, ${\hat{y}}_{ic}$ represents the predicted probability of the *i*-th sample belonging to the *c*-th class, *n* is the total number of samples, and *N* is the number of classes in the classification problem.
  (c) F1 score, defined as F1 = 2 (precision × recall)/(precision + recall)

A summary of the hyperparameters used is shown in Table 2. This architecture provides a robust framework for image classification tasks, offering flexibility for experimentation and optimization to achieve high accuracy and efficiency.

Upon loading the data, the input images and their corresponding labels were extracted. The labels were structured according to a predefined mapping, allowing for clear identification and categorization of the data.

In preparation for model training and evaluation, the dataset was partitioned into training and testing subsets. This partitioning was achieved with

- a training size of 80% ($N_{\mathrm{train}}$ = 14,640 images), with a validation split of 10%;
- a test size of 20% ($N_{\mathrm{test}}$ = 36,600 images);
- a fixed random state for reproducibility.

The resulting training set comprises a majority of the data, utilized for model training and parameter optimization. Conversely, the testing set served as an independent dataset for assessing the generalization performance of the trained models. This separation into distinct training and testing sets facilitated unbiased estimation of the model’s predictive capabilities on unseen data, essential for gauging its applicability and robustness.

## 3. Results

**Figure 6:**
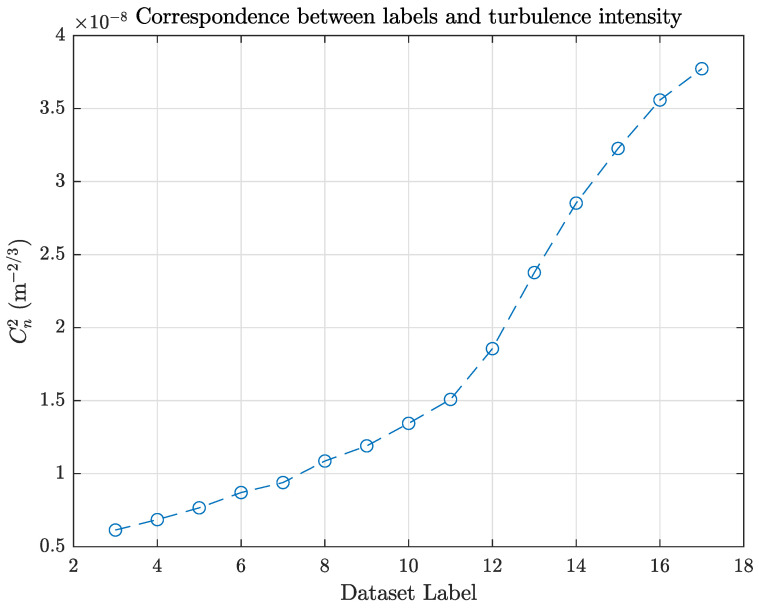
Correspondence between the assigned labels and the turbulence intensity values.

To capture data, two control experiments were conducted initially, one with the turbulator completely off and the other with only the fans on. They were labeled ‘01’ and ‘02’, respectively, and were not considered for the categorical analysis of this work since they do not include turbulence information; therefore, the categories used for this analysis were ‘03’, …, ‘17’, representing increasing turbulence intensity. The equivalence between the labels used and the intensity of the turbulence is shown in Table 1 and Figure 6.

**Figure 7:**
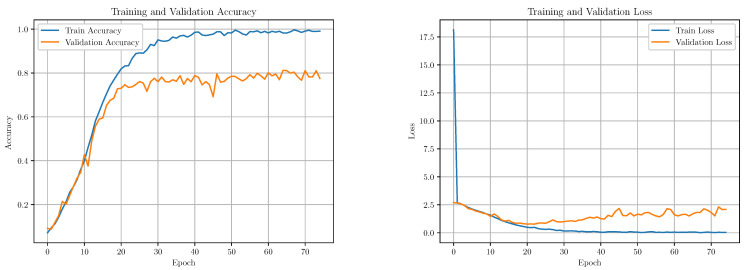
Model training performance. (**Left**) Training accuracy; (**right**) training loss.

We conducted an analysis of the training performance of our model by monitoring key metrics such as accuracy and loss throughout the training process (Figure 7). To gain insights into the model’s learning dynamics and generalization capabilities, we plotted the accuracy and loss for both the training and validation datasets versus epochs. These plots provide a comprehensive view of the model’s performance over time, allowing us to observe how it evolves and adapts to the training data while also assessing its ability to generalize to unseen data.

## 4. Discussion

**Figure 8:**
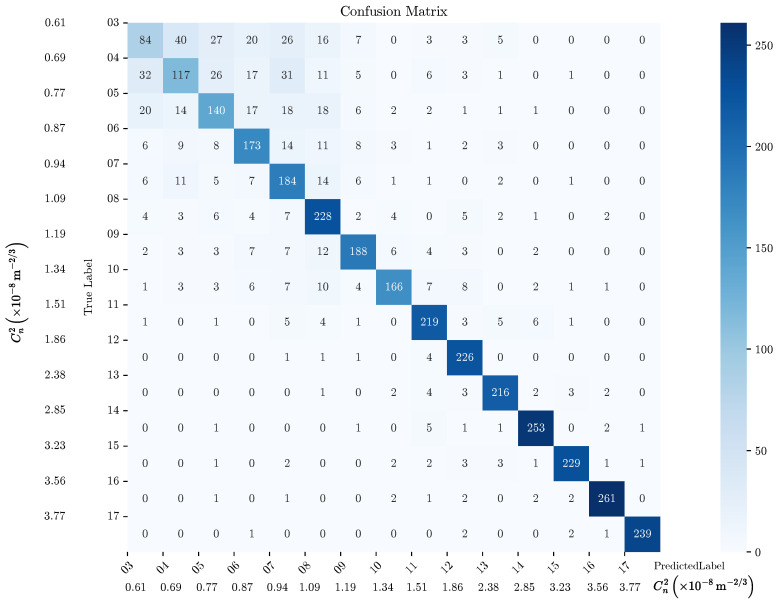
Confusion matrix.

To evaluate the model’s performance, we employed a confusion matrix on the test dataset (Figure 8). The confusion matrix is a tabular representation of the performance of a classification model, where each row of the matrix represents the instances in a predicted class, and each column represents the instances in an actual class. Each element ($M_{ij}$) of the matrix represents the number of instances known to be in class (*i*) and predicted to be in class (*j*). To calculate the confusion matrix, we compared the true labels ($y_{\mathrm{true}}$) with the predicted labels ($y_{\mathrm{pred}}$). We incremented the count in ($M_{ij}$) if the true label was (*i*) and the predicted label was (*j*).

**Figure 9:**
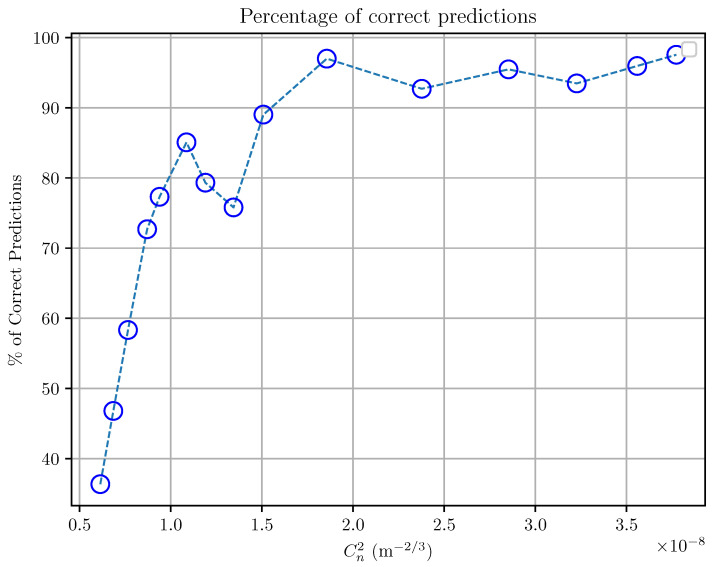
Percentage of correct predictions for the different turbulence intensity values.

This matrix provided a detailed breakdown of the correct and incorrect predictions for each label (turbulence intensity). Focusing on the diagonal elements of the matrix, which represent the correctly classified samples, we plotted them as a percentage of correct predictions versus the intensity of the turbulence (Figure 9). This analysis revealed that for turbulence intensities ($C_{n}^{2}$) greater than or equal to $1.5\times 10^{-8}$ m^−2/3^, the model achieved a success rate exceeding 90%. This suggests that the model performs very well in identifying higher turbulence intensities.

This highlights the robustness of the proposed approach in detecting more pronounced turbulence scenarios, likely due to the distinctive spatio-temporal patterns that such intensities produce in the video slices.

In contrast, the model exhibited relatively lower accuracy for lower turbulence intensities (lower than $1.5\times 10^{-8}$ m^−2/3^), where the distinguishing features in the spatio-temporal domain were subtler and potentially more influenced by noise. This behavior shows the challenge of discerning fine-grained variations in low-intensity turbulence and suggests opportunities for further refinement in data preprocessing or feature extraction to enhance sensitivity to minor perturbations in future work.

The results depicted in Figure 9 also suggest the potential of this methodology for real-world applications, as it demonstrates reliability in scenarios where the accurate characterization of higher turbulence is crucial, such as in free-space optical communication or astronomical imaging. The practical implications include the ability to achieve reliable measurements with limited equipment, paving the way for broader accessibility and the deployment of turbulence quantification tools.

Further investigations could focus on enhancing the model’s performance in low-intensity turbulence conditions by incorporating alternative deep learning architectures or utilizing higher-resolution input data. Additionally, extending the analysis to include temporal variations in classification confidence might provide deeper insights into the dynamics of atmospheric turbulence across different intensity ranges.

The integration time of each captured frame in our study was approximately 1/24 s, which is relatively long compared to previous high-speed turbulence studies, such as Ref. [30], where shorter exposure times (1/500 s) were used to analyze fast dynamic turbulence fluctuations. However, in our previous works (Refs. [7,8]), we demonstrated that images obtained with integration times similar to the one used in this study still retain essential information about turbulence intensity, provided that the number of temporal samples is sufficiently large. Determining the minimum necessary integration time (in terms of frames) for ensuring reliable turbulence quantification is indeed an interesting problem, and we recognize it as a key point for future research. In subsequent studies, we aim to explore how variations in integration time may affect the ability of deep learning models to extract turbulence-related features, ultimately refining the method for broader applications.

The key metrics are summarized in Table 3.

### 4.1. Training Set Considerations and Generalization

One of the key concerns in deep learning models for turbulence quantification is their ability to generalize beyond the specific conditions under which they were trained. Since our dataset is derived from a controlled experimental setup with a well-characterized turbulator, there is a possibility that the model may exhibit overfitting to the statistical properties of turbulence specific to this setup.

To enhance generalization, we considered applying data augmentation techniques to artificially expand the training set (mirroring and flipping images), which introduces additional variations without altering the fundamental turbulence characteristics. However, our results show that the dataset, as described in this paper, already yields good classification performance, suggesting that the trained model effectively captures relevant turbulence features, even without a limited augmentation strategy.

An alternative approach to enhance generalization could involve modifying the Region of Interest (ROI) dynamically. For example, extracting a smaller ROI and applying gradual shifts across frames could introduce new variations that simulate atmospheric turbulence effects on dynamic target imaging. This technique may be particularly useful in understanding how turbulence affects moving targets, such as airborne objects or surveillance applications. While this direction is beyond the scope of the current study, it presents an interesting avenue for future work, where incorporating dynamic spatial shifts in dynamic targets could contribute to improved model adaptability in real-world conditions.

### 4.2. Impact of Non-Uniform Turbulence on Quantification

In real-world atmospheric conditions, turbulence is rarely uniform along an optical propagation path. Instead, it varies due to environmental factors such as altitude-dependent temperature gradients, etc. This variation influences the refractive index structure constant, $C_{n}^{2}$, which is commonly integrated along the path to obtain a path-weighted turbulence strength metric. Our current experimental setup employs a turbulator to create controlled and statistically repeatable turbulence conditions, assuming a uniform turbulence layer over the short propagation path. However, in practical applications, turbulence is often distributed in layers of varying intensities and distances from the imaging system, leading to different effects on optical wavefronts. To extend the applicability of our method, the following would be it would be necessary:

- Incorporate a turbulence weighting function that accounts for distance-dependent effects. This could be achieved using known optical turbulence models that integrate $C_{n}^{2}$ along the path.
- Train the model in environments where turbulence is layered or varies dynamically, ensuring that the trained neural network can generalize beyond the uniform turbulence conditions created in the controlled laboratory experiment. This, in principle, may be achieved (a) with a set of turbulators in series, each one functioning with the appropriate settings (as suggested in [33]), or (b) by imaging in open field, taking measurements of local turbulence intensity along the propagation path with specialized equipment.

Several practical applications involve turbulence profiles that are highly non-uniform [1]:

- Free-Space Optical (FSO) Communication: In long-range optical links, turbulence is typically strongest near the ground and weaker at higher altitudes. The impact of turbulence on signal quality depends on whether it occurs at the transmitter, mid-path, or receiver.
- Astronomical Imaging: The Earth’s atmospheric boundary layer contributes significantly to image degradation in ground-based telescopes, while higher-altitude turbulence can be weaker but still distorts wavefronts.
- Remote Sensing and Surveillance: Optical systems imaging distant objects often experience turbulence concentrated in specific regions, such as near urban heat sources or over bodies of water.

These applications suggest that adapting our deep learning model to account for non-uniform turbulence distributions could be valuable for real-world deployments. Future work will involve training the model on synthetic or real data where turbulence strength is weighted based on its position along the optical path.

## 5. Conclusions

Our approach offers several advantages. First, it leverages the affordability and accessibility of video cameras, making turbulence measurements more cost-effective and widely deployable versus specialized equipment approaches. In addition, space-time analysis captures the dynamic nature of turbulence, providing a more comprehensive understanding than single-point measurements.

We evaluated the performance of our proposed method on real turbulence data, demonstrating its accuracy and generalizability in quantifying turbulence intensity across different experimental conditions. Our findings showcase the potential of deep learning and video analysis in advancing atmospheric turbulence characterization, opening the doors for improved applications in various fields. Although the values of $C_{n}^{2}$ are discretized in this technique, good sampling was enough in the training and subsequent use of the deep learning model.

These findings have significant real-world implications, particularly for imaging applications where turbulence characterization is critical. The turbulator’s characterization in terms of $C_{n}^{2}\Delta h$, where Δh represents the propagation distance, suggests that this approach can effectively be applied to lower turbulence intensities over extended distances (provided the single uniform layer hypothesis is fulfilled).

Additionally, the deep learning-based method presented in this paper offers several advantages that may benefit real world applications:

- Scalability for real-time applications: Once trained, the trained model can infer turbulence levels in practically real-time, making it suitable for adaptive optics and free-space optical communication systems. The trained model’s compact size (∼41 MB) facilitates easy deployment, while the ability to achieve accurate characterization with short video captures (approximately 225 frames, or 9 s) makes this method practical for real-world use.
- Does not require manual analysis.
- No reliance on explicit optical models: Unlike, for example, Shack–Hartmann or AoA methods, our technique does not require an analytical turbulence model. In fact, this method is agnostic with respect to the turbulence model used to provide the turbulence intensity value associated with each set of training images (in this case, the AoA method used to characterize the turbulator). This makes the model easily adaptable to other turbulence models on real or simulated data.
- Ability, in principle, to handle complex turbulence regimes: Traditional methods assume Kolmogorov turbulence, while CNNs can adapt to a variety of turbulence patterns under any proposed turbulence regime, real or simulated.
- We evaluated the performance of our proposed method, obtaining high accuracy in quantifying turbulence intensity, particularly for values exceeding $1.5\times 10^{-8}$ m^−2/3^.

The proposed deep learning-based turbulence quantification method has potential for real-time monitoring in free-space optical communication, where rapid turbulence assessment can enhance signal integrity. Furthermore, turbulence mitigation in imaging systems may benefit from this rapid determination of $C_{n}^{2}$, paving the way for deployment in adaptive optics in astronomical imaging and wavefront sensing in laser beam propagation.

Considering practical scenarios such as free-space laser communications, it is possible to implement this method by simply observing a static target. This approach will be addressed in future work as it requires previous extensive laboratory training of this model in terms of $C_{n}^{2}\cdot \Delta h$, where Δh is the propagation distance [29]. Future efforts will focus on the enhancement of lower turbulence intensities for this methodology, the incorporation of color information, analyzing evolving image sequences, and determining the minimum capture duration required for optimal performance.

## Figures and Tables

**Table 1:** Label correspondence to temperature difference and turbulence intensity.

Label	ΔT (C)	$C_{n}^{2}$ ($10^{-8}$ m^−2/3^)
03	2.60	0.61
04	5.70	0.69
05	8.90	0.77
06	12.70	0.87
07	15.00	0.94
08	19.60	1.09
09	22.60	1.19
10	26.80	1.34
11	31.00	1.51
12	39.40	1.86
13	51.70	2.38
14	64.00	2.85
15	75.90	3.23
16	90.70	3.56
17	106.20	3.77

**Table 2:** Hyperparameters used in the CNN model.

Hyperparameter	Value Used	Notes
Input Shape	(225, 225)	Input image size
Convolutional Layers	3 Conv2D layers	8, 16, and 32 filters with (3 × 3) kernels
Activation Function	ReLU (Conv & Dense), Softmax (Output)	ReLU prevents vanishing gradients
Stride	(2, 2) in all Conv2D layers	Downsampling
Padding	Same	Ensures feature preservation
Flatten Layer	Present	Converts feature maps to a dense representation
Dense Layers	2 (128 units + output layer)	128-unit FC layer before final classification
Output Classes	15	Based on label map categories
Optimizer	Adam	Common choice for CNNs
Loss Function	Categorical Cros-sentropy	Standard for multi-class classification
Batch Size	32	
Epochs	75	
Validation Split	10%	Used during training
Regularization	None	
Cross Validation	None	
Weight Initialization	Default (Keras)	
Data Augmentation	Mirroring, Flipping	None applied

**Table 3:** Summary of training metrics. Label correspondence to turbulence intensity is shown in Figure 6.

Label	Precision	Recall	F1-Score	Support
03	0.54	0.36	0.43	231
04	0.58	0.47	0.52	250
05	0.63	0.58	0.61	240
06	0.69	0.73	0.71	238
07	0.61	0.77	0.68	238
08	0.70	0.85	0.77	268
09	0.82	0.79	0.81	237
10	0.88	0.76	0.82	219
11	0.85	0.89	0.87	246
12	0.85	0.97	0.91	233
13	0.90	0.93	0.92	233
14	0.94	0.95	0.95	265
15	0.95	0.93	0.94	245
16	0.97	0.96	0.96	272
17	0.99	0.98	0.98	245
Accuracy			0.80	3660
Macro avg	0.79	0.80	0.79	3660
Weighted avg	0.80	0.80	0.79	3660

## Data Availability

The data used in this manuscript are available upon request.
